# Variation of global DNA methylation levels with age and in autistic children

**DOI:** 10.1186/s40246-016-0086-y

**Published:** 2016-09-23

**Authors:** Shui-Ying Tsang, Tanveer Ahmad, Flora W. K. Mat, Cunyou Zhao, Shifu Xiao, Kun Xia, Hong Xue

**Affiliations:** 1 Division of Life Science, Applied Genomics Centre and Centre for Statistical Science, Hong Kong University of Science and Technology, Clear Water Bay, Hong Kong, China; 2 Department of Medical Genetics, School of Basic Medical Science, Southern Medical University, Guangzhou, Guangdong 510515 China; 3 Department of Geriatric Psychiatry, Shanghai Mental Health Center, Shanghai Jiaotong University School of Medicine, Shanghai, 200030 China; 4 The State Key Laboratory of Medical Genetics, Central South University, Changsha, Hunan 410078 China

**Keywords:** Aging, Autism spectrum disorder, CpG methylation, Developmental epigenetics, Genome-wide methylation quantification

## Abstract

**Background:**

The change in epigenetic signatures, in particular DNA methylation, has been proposed as risk markers for various age-related diseases. However, the course of variation in methylation levels with age, the difference in methylation between genders, and methylation-disease association at the whole genome level is unclear. In the present study, genome-wide methylation levels in DNA extracted from peripheral blood for 2116 healthy Chinese in the 2–97 age range and 280 autistic trios were examined using the fluorescence polarization-based genome-wide DNA methylation quantification method developed by us.

**Results:**

Genome-wide or global DNA methylation levels proceeded through multiple phases of variation with age, consisting of a steady increase from age 2 to 25 (*r* = 0.382) and another rise from age 41 to 55 to reach a peak level of ~80 % (*r* = 0.265), followed by a sharp decrease to ~40 % in the mid-1970s (age 56 to 75; *r* = −0.395) and leveling off thereafter. Significant gender effect in methylation levels was observed only for the 41–55 age group in which methylation in females was significantly higher than in males (*p* = 0.010). In addition, global methylation level was significantly higher in autistic children than in age-matched healthy children (*p* < 0.001).

**Conclusions:**

The multiphasic nature of changes in global methylation levels with age was delineated, and investigation into the factors underlying this profile will be essential to a proper understanding of the aging process. Furthermore, this first report of global hypermethylation in autistic children also illustrates the importance of age-matched controls in characterization of disease-associated variations in DNA methylation.

**Electronic supplementary material:**

The online version of this article (doi:10.1186/s40246-016-0086-y) contains supplementary material, which is available to authorized users.

## Background

Genetic changes can alter the genomic DNA sequence through point mutations, insertions, deletions, copy number variations, and chromosomal rearrangements while epigenetic modifications can modulate phenotype and gene expressions. DNA methylation is the most common epigenetic modification that plays an essential role in the regulation of tissue-specific gene expression, cellular differentiation, chromosome stabilization, genomic imprinting, and suppression of transposable element mobility [1, 2]. DNA methylation through DNA methyltransferases convert cytosine to 5-methycytocine, with the majority of the conversions occurring at CpG islands found in gene promoter regions. Aberrant DNA methylation patterns have long been associated with various human diseases including cancers, cardiovascular diseases, psychotic disorders, and autism [3–6].

Changes in epigenetics signatures, and in particular DNA methylation, have been reported to occur in normal physiological development and aging, and alterations in DNA methylation associated with the signaling and regulation of transcription have been demonstrated in some genes [7, 8]. Aging is the gradual deterioration of various body functions and represents an important risk factor for various age-related diseases such as cancer, neurodegenerative disorders, cardiovascular diseases, and type 2 diabetes mellitus [9]. Several studies have examined DNA methylation changes in old age as disease risk factor, focusing mostly on CpG islands and promoter regions in specific gene [10, 11]. However, the characterization of lifelong age-related changes in DNA methylation at the whole genome level has remained a largely unexplored area.

During the past decades, various HPLC-based, sequencing-based, (e.g., bisulfite-sequencing and methylated DNA immunoprecipitation) and microarray-based methods have been introduced to quantitate genomic DNA methylation [12]. Although these methods enable high-resolution and detailed methylation profiles of individual genes, they are time-consuming and incapable of measuring whole genome methylation levels accurately. Recently, a number of methods have been developed to render possible the measurement of whole genome methylation levels, including the LUminometric Methylation Assay (LUMA) method [13], the ELISA-based approach [14], and the fluorescence polarization DNA methylation (FPDM) method developed by us [15].

The objective of the present study was to analyze whole genome DNA methylation in the normal population in order to establish the quantitative relationship between global DNA methylation levels and age using the simple and accurate FPDM method, as well as delineate any gender differences. The DNA methylation-aging curve obtained for the normal population will provide a useful reference to facilitate an improved understanding of the regulation of DNA methylation in aging. Moreover, autism-associated changes in genome-wide methylation were investigated, which also served to demonstrate the importance of using age-matched controls in methylation-disease association studies.

## Methods

### Study population

The main study population in this study were enrolled from Beijing, Shanghai, Changsha, and Hong Kong and consisted of 2116 healthy Chinese subjects including 1108 (52.36 %) males and 1008 (47.64 %) females. The subjects spanned a wide age range from 2 to 97 years. The 280 autistic children (age 2-13 years) and their parents (*n* = 552; age 24–62 years) were recruited at Central South University in Changsha. Samples from the parents but not those from the children were included in the main study set for age-methylation analysis.

### Genomic DNA extraction

Leukocytes were isolated from 5-ml peripheral blood samples. DNA was prepared by phenol extraction and chloroform extraction followed by isopropanol precipitation, washed with ethanol, and air-dried. Tris-EDTA buffer pH 8.0 was used to dissolve the final genomic DNA product.

### Whole genome DNA methylation analysis by FPDM

To determine genome-wide or “global” DNA methylation by fluorescence polarization DNA methylation measurement, ~100 ng DNA sample was first subjected to separate restriction-enzyme digestions by HpaII and MspI as described [15]; the methylation-sensitive HpaII cut only un-methylated 5′CCGG-3′ sites, whereas the methylation-insensitive MspI cut both methylated and un-methylated 5′-CCGG-3′ sites. After completion of the restriction reactions, both digests were subjected to a one-label-extension reaction through incubation with fluorescent TAMRA-dCTP (5-propargylamino-dCTP-5/6-carboxytetra-methylrhodamine, Jena Bioscience) and Taq DNA polymerase. Measurements of fluorescence polarization on the two digests following the extension reaction yielded the percentile global methylation in the DNA sample. The global DNA methylation level in each instance was thus expressed in terms of the global percentage of CpG sites in genomic DNA that were methylated based on the average of triplicate measurements.

### Statistical data analysis

Statistical analysis of data was performed using SPSS 19.0. Percentile methylation of each DNA sample represented the average of three independent measurements. To assess the relationship between global DNA methylation and age, methylation levels of samples in every 5-year age range were first analyzed for correlation with age using Pearson’s correlation test. Based on these results, the samples were further grouped into five age ranges to represent different phases of methylation change with age and again analyzed using Pearson’s correlation to yield an overall correlation coefficient for each age range. Differences in methylation levels between males and females were analyzed for all samples using independent sample *t* test as well as for each group of samples in the five age ranges using multivariable linear regression. Independent sample *t* test was used to analyze the methylation difference between autistic children and parents and between autistic children and age-matched healthy children. A *p* value <0.05 was regarded as statistically significant.

## Results

**Fig. 1:**
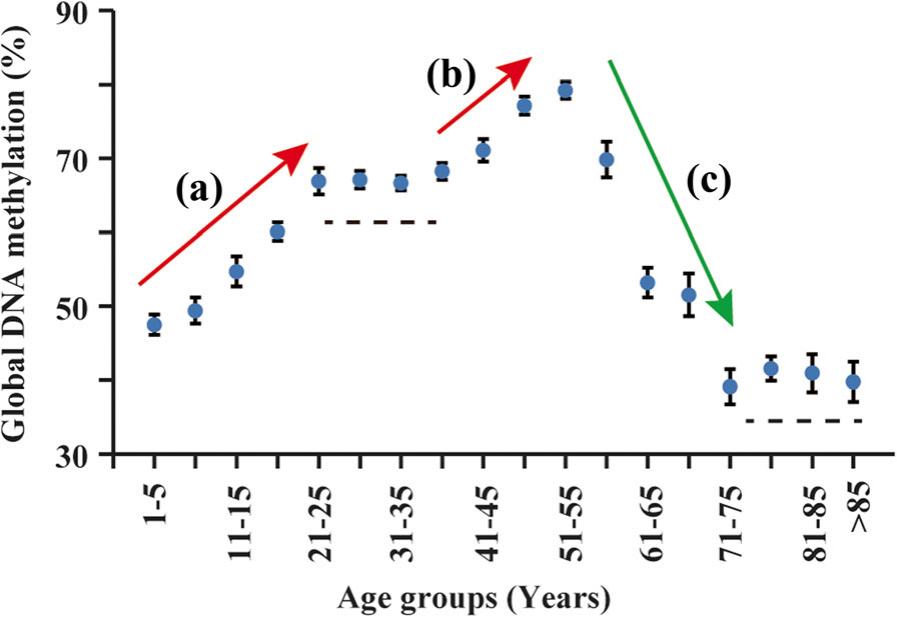
Variation of global DNA methylation with age. Mean global DNA methylation of 5-year age groups are shown. *Error bars* indicate the standard errors of the mean, and the three *arrows* represent (*a*) increasing trend at age range 1–25 years, (*b*) increasing trend at age range 40–55 years, and (*c*) decreasing trend at age range 55–75 years. The two *dashed lines* indicate the two age ranges (25–40 and over 75 years) where there were no significant changes in methylation

The methylation data is given in Additional file 1: Table S1. Although the global DNA methylation data determined using the FPDM method displayed large standard deviations, when the subjects were divided into 5-year age groups and analyzed for within-group correlations with age, positive correlations were detected in the 16–20 and 51–55 groups, and a negative correlation was detected in the 56–60 group (Table 1). Based on the within-group correlations and the methylation-age plot (Fig. 1), multiple phases of change in methylation levels with age were discerned including a steady increase from year 2 to year 25 and another rise from year 40 onward to reach a peak level at year 55, followed by a sharp decrease up to year 75 and leveling off thereafter. Quantitatively, the increase from age 2 up to the age of 25 was significant to *p* < 0.001 with Pearson’s correlation coefficient *r* = 0.382. From 26 to 40 years of age, there was no significant change in methylation (*r* = 0.028; *p* = 0.459). However, the DNA methylation levels again significantly increased with age between 41 to 55 years (*r* = 0.265; *p* < 0.001). From 56 to 75 years of age, there was a significant decrease in global DNA methylation (*r* = −0.395; *p* < 0.001) showing an inverse relationship between methylation and age, and no significant change in methylation levels was observed between 75 and 97 years of age (*r* = −0.061; *p* = 0.486). The correlation data for these different age groups are given in Additional file 2: Table S2.

**Table 1:** Global DNA methylation levels of different age groups

Age range	Number	Global DNA methylation (%, mean ± SD)	Pearson’s *r* ^a^	*p* value^b^
1–5	160	47.52 ± 17.51	0.040	0.614
6–10	93	49.41 ± 17.03	0.053	0.613
11–15	68	54.73 ± 16.69	0.055	0.658
16–20	207	60.10 ± 17.78	0.205	*0.003*
21–25	100	66.92 ± 17.78	0.029	0.775
26–30	188	54.11 ± 16.10	0.008	0.916
31–35	321	66.68 ± 17.95	−0.059	0.292
36–40	202	68.24 ± 15.96	0.127	0.073
41–45	112	71.09 ± 16.08	0.150	0.113
46–50	129	77.17 ± 14.13	0.138	0.120
51–55	92	79.22 ± 10.95	0.213	*0.041*
56–60	68	69.84 ± 20.02	−0.294	*0.015*
61–65	120	53.20 ± 22.13	−0.028	0.765
66–70	71	51.54 ± 24.41	−0.061	0.612
71–75	54	39.13 ± 17.18	−0.133	0.339
76–80	75	54.73 ± 16.69	−0.064	0.584
81–85	36	40.95 ± 15.27	−0.023	0.896
>85	20	39.78 ± 12.08	−0.073	0.758

^a^Pearson’s correlation coefficients pertaining to within-group analysis

^b^ *p* values less than 0.05 are shown in italic font

With respect to the genders (Table 2), a statistically significant gender effect was observed in the 41 to 55 age group (beta = 0.136; *p* = 0.010), where the average methylation levels in males (73.49 ± 15.26) was higher than that in females (77.52 ± 13.44). There was no significant gender effect in any of the other age groups or when all age groups were combined.

**Table 2:** Global DNA methylation levels in different male and female age groups

Age groups	Gender	Number	Global DNA methylation (%, mean ± SD)	*p* value^a^
2–25	Male	414	54.99 ± 18.70	0.370
	Female	214	57.42 ± 18.92	
26–40	Male	356	67.17 ± 16.56	0.816
	Female	355	67.31 ± 17.29	
41–55	Male	151	73.49 ± 15.26	*0.010*
	Female	182	77.52 ± 13.44	
56–75	Male	127	56.42 ± 23.34	0.152
	Female	186	52.37 ± 23.50	
75–97	Male	60	40.72 ± 14.14	0.711
	Female	71	41.46 ± 14.20	
Overall	Male	1108	60.81 ± 19.86	0.060^b^
	Female	1008	62.47 ± 20.81	

*p* < 0.05 is shown in italic font

^a^ *p* values using gender as a variable in multivariable linear regression analyses

^b^ *p* value for between-gender comparison using Student’s *t* test

**Fig. 2:**
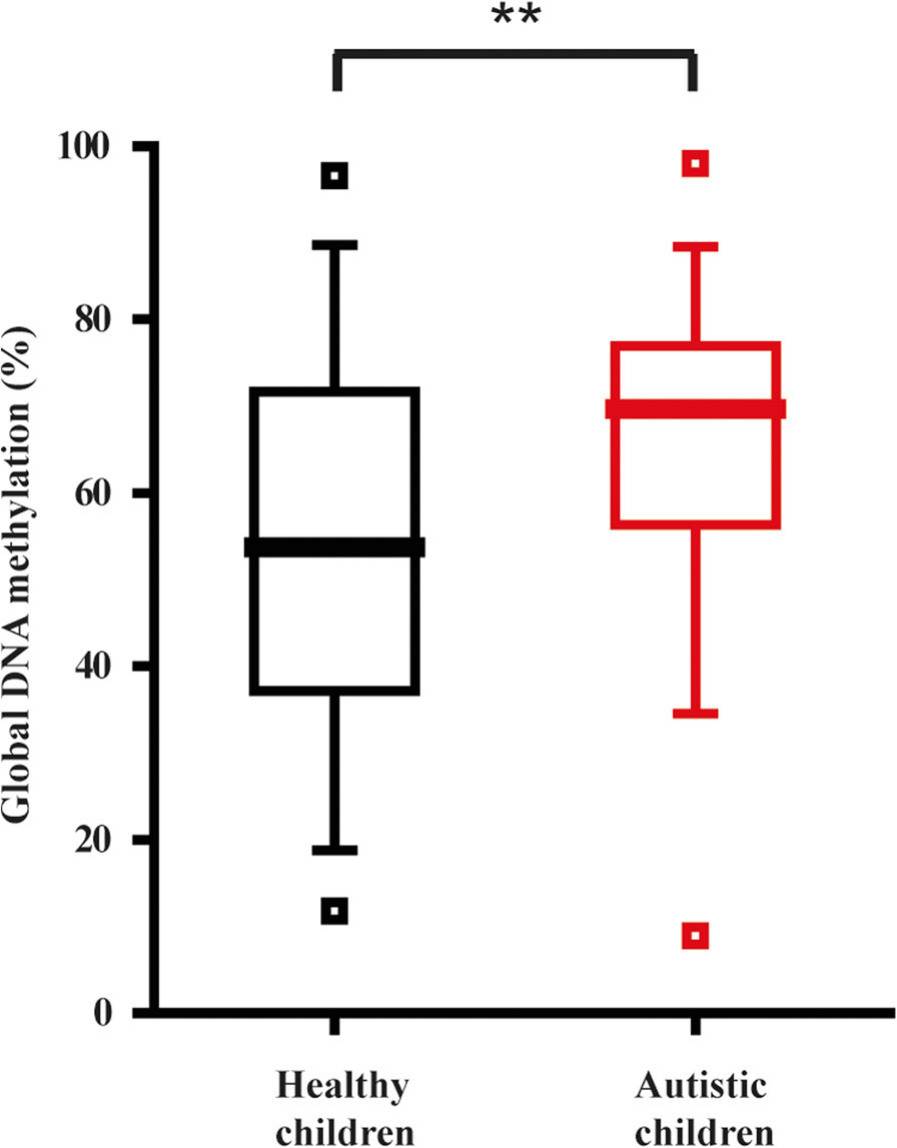
Variation of global DNA methylation in autistic children. The *box plot* shows methylation levels in healthy children (*black*) and autistic children (*red*). The *horizontal line* in each box indicates the median; the *upper* and *lower bounds* of the box represent the 75th and 25th percentiles, respectively; the *upper* and *lower whiskers* for each box mark the 95th and 5th percentiles, respectively; and the *squares* indicate the maximum and minimum values. Double asterisk indicates *p* < 0.001 for the pairwise comparison under the independent sample *t* test

The global methylation levels for autistic children (*n* = 280; mean age = 4.7) were compared to both those of their healthy parents (*n* = 552; mean age = 33.8) and those of age-matched healthy children (*n* = 236; mean age = 5.3). No significant difference (*p* = 0.872) was observed between patients (65.18 ± 16.69) and parents (66.01 ± 19.98) (Additional file 3: Table S3); but the difference between patients and age-matched controls (54.35 ± 21.37) was highly significant (*p* < 0.001), with increased methylation in the patients (Fig. 2). There was no significant difference in methylation between autistic children and either their fathers or mothers separately (Additional file 3: Table S3).

## Discussion

The purpose of the present study has been to analyze, using the FPDM method, the global DNA methylation levels in leukocytes as a function of age in order to establish a continuous methylation-age curve for the population that could serve as a basis for describing phenotypic changes associated with aging and as an age-dependent standard for the detection of any significant deviation caused by disease. In providing a systematic characterization of the dependence of global DNA methylation on age, the study revealed that global DNA methylation as a genomic parameter of age was distinctly multiphasic in character. The global DNA methylation-age curve displayed evident increases over the adolescent age group of 10–20 and late middle-age age group of 40–50 and a sharp decrease over the age group of 55–70 to reach a hypomethylation level from age 71 onwards as a hallmark of old age. Previous studies on age-associated global DNA methylation have reported age-dependent decrease in methylation based on adult to old age populations. In addition, there are also reports of age-associated increase in methylation [7] and methylation increases in the early years of life [16]. Moreover, while many loci such as intergenic CpGs outside of CpG islands display decreased methylation in later life, other loci such as promoter-associated CpG islands show increased methylation with age throughout the lifespan [8]. Therefore, in general global methylation decreases in old age, it has not been established that this decrease occurs continuously throughout life. Indeed, a general increase in DNA methylation with age before adulthood, followed by stabilization and an eventual decrease in old age, has emerged from studies on different age ranges using different methods [8]. The lifelong profile obtained in the present study is consistent with this general description, with the more comprehensive time curve revealing a second period of methylation increase in late middle-age prior to the methylation decline in old age.

Significant difference between male and female subjects was observed only in the late middle-age age group, suggesting that gender-related factors may contribute to this second period of methylation increase. The profile of global methylation variation between genders is somewhat unclear with previous reports of significant difference based on methylation levels in LINE-1 repeat elements for a 45–75 age group [17] but no difference for a 19–80 age group [18] and no significant difference based on the LUMA method for CCGG sites for a group with mean age of 24 [19]. The different results suggest that gender differences in “global” methylation levels are dependent on age as well as the subset of methylation sites examined in the quantification method.

DNA hypomethylation at old age has been reported in studies focused on a relatively limited number of gene loci and narrow age ranges, suggesting the possible association between DNA methylation and age-related diseases [9, 20, 21]. Indeed, the loss of global DNA methylation is one of the first epigenetic abnormalities in cancers [6], and advanced age represents a potent risk factor for human epithelial cancers with cancer incidence increasing sharply from age 60 onward, especially in males [22], which is in accord with the sharp decrease in DNA methylation over this age range shown in Fig. 1. Likewise, evidence of age-associated loss of DNA methylation in brain tissue suggests the significant role of DNA hypomethylation at old age in the pathogenesis of Alzheimer’s disease [23]. The onset of Alzheimer’s disease at age 65 is also in accord with the sharp decrease in global DNA methylation between the ages 55–70 shown in Fig. 1.

Unlike neurodegenerative and aging-related disorders, neurodevelopmental disorders such as autism and Down syndrome affect subject groups on the opposite end of the age spectrum. Autism is highly heritable and affects information processing in the brain, leading to symptoms that include impairments in social interaction and communication, restricted interest, and repetitive behavior [24]. These characteristic symptoms become apparent in early childhood, typically before the age of three. Although the etiology of autism is mainly ascribed to genetic variations including single nucleotide polymorphisms and copy number variations [25], epigenetic mechanisms have been invoked to affect the environmental influences [4, 26]. As such, DNA methylation has been associated with dysregulation of biological pathways in autistic brains, with both hypomethylated and hypermethylated genomic regions being identified [5]. Recently, in peripheral blood analysis, the *OXTR* promoter was shown to be hypomethylated in autism cases [27]. Here, we have demonstrated that the global methylation in autistic children was increased compared to healthy children (Fig. 2) with respect to the overall effect across all CCGG sites recognized by the HpaII/MspI enzymes, encompassing both hypermethylated and hypomethylated sites as well as unchanged sites. The overall increase suggests that hypermethylated regions were more extensive than hypomethylated regions in the autistic genome. Moreover, in comparison with the time profile for methylation, the higher methylation level is that expected of young to middle-aged adults and this could be interpreted to suggest an abnormally advanced methylome in autistic children. This is reflected in that no significant difference in methylation was found between autistic children and their parents. From another point of view, since a general increase in methylation takes place from young to middle age (Fig. 1), the comparison between children and parents is confounded by the age factor, and the result demonstrates the importance of using age-matched controls in analyzing methylation differences. Notably, environmental and nutritional factors may also affect methylation, and application of the FPDM method will facilitate the in-depth analysis of the quantitative effects of such external factors.

In conclusion, global DNA methylation measurements in the present study on leukocyte DNA have shown a multiphasic variation with age that leads to depression of methylation at old age to half its level at middle age, thereby providing strong evidence for DNA hypomethylation at old age as a potent risk marker for various age-related disorders such as cancers, cardiovascular and neurodegenerative disorders, and type 2 diabetes. In contrast, hypermethylation was observed for autism, a neurodevelopmental condition. These measurements, readily performed with the FPDM method, provide a simple and quantitative approach to investigate the multiple genetic and environment factors that determine global DNA methylation. A delineation of global methylation changes will complement studies on gene-specific methylation changes to yield an increasingly comprehensive understanding of the regulation of DNA methylation and the roles of DNA methylation in age-related diseases.

## Additional files

Additional file 1: Table S1. Sample information and methylation data. (XLSX 118 kb) Additional file 2: Table S2. Correlation of global DNA methylation with age. (DOCX 13 kb) Additional file 3: Table S3. Global DNA methylation levels of autistic children and their parents. (DOCX 12 kb)

## Abbreviation

FPDM: Fluorescence polarization DNA methylation
