# Supplementary material for: Variation of global DNA methylation levels with age and in autistic children
Source: Hum Genomics. 2016 Sep 23;10:31. doi: 10.1186/s40246-016-0086-y (PMC5035466; doi:10.1186/s40246-016-0086-y)
Supplement: Additional file 2: Table S2. — Correlation of global DNA methylation with age. (DOCX 13 kb) [file 40246_2016_86_MOESM2_ESM.docx]

**Table S2.** Correlation of global DNA methylation with age

| Age Groups | N | Global DNA methylation  (%, Mean ± SD) | Pearson’s r ^a^ | *p* value ^b^ |
| --- | --- | --- | --- | --- |
| 2-25 | 628 | 55.82 ± 18.80 | 0.382 | **<0.001** |
| 26-40 | 711 | 67.24 ± 16.92 | 0.028 | 0.459 |
| 41-55 | 333 | 75.69 ± 14.41 | 0.265 | **<0.001** |
| 56-75 | 313 | 54.01 ± 23.48 | -0.395 | **<0.001** |
| 75-97 | 131 | 41.12 ± 14.12 | -0.061 | 0.486 |

^a^ Pearson’s correlation coefficients (r) pertaining to within group linear regression analysis

^b^ *p* values less than 0.05 are shown in bold font
