# Supplementary material for: Variation of global DNA methylation levels with age and in autistic children
Source: Hum Genomics. 2016 Sep 23;10:31. doi: 10.1186/s40246-016-0086-y (PMC5035466; doi:10.1186/s40246-016-0086-y)
Supplement: Additional file 3: Table S3. — Global DNA methylation levels of autistic children and their parents. (DOCX 12 kb) [file 40246_2016_86_MOESM3_ESM.docx]

**Table S3.** Global DNA methylation levels of autistic children and their parents

| Group | N | Mean age | Global DNA methylation  (%, Mean ± SD) | *p* value ^a^ |
| --- | --- | --- | --- | --- |
| Autistic children | 280 | 4.7 | 65.81 ± 16.69 | - |
| Parents | 552 | 33.8 | 66.01 ± 16.98 | 0.872 |
| Fathers | 278 | 35.0 | 65.89 ± 17.23 | 0.967 |
| Mothers | 274 | 32.6 | 66.15 ± 16.75 | 0.811 |

^a^ *p* value for comparison between autistic children and the respective group using independent samples t-test
